# Effects of *Litsea cubeba* Essential Oil–Chitosan/Corn Starch Composite Films on the Quality and Shelf-Life of Strawberry (*Fragaria* × *ananassa*)

**DOI:** 10.3390/foods13040599

**Published:** 2024-02-16

**Authors:** Hongjun Fu, Liyuan Wang, Jiahui Gu, Xianglian Peng, Jian Zhao

**Affiliations:** 1 College of Food Science and Engineering, National Engineering Laboratory for Deep Process of Rice and Byproducts, Central South University of Forestry and Technology, Changsha 41004, China; hongjun4648@163.com (H.F.); 18534369137@163.com (L.W.); gujiahui0822@163.com (J.G.); 2 Food Science and Technology, School of Chemical Engineering, UNSW Australia, Sydney 2052, Australia

**Keywords:** chitosan, *Litsea cubeba* essential oil, strawberry, composite coating

## Abstract

In this work, we have developed a composite chitosan film incorporating the *Litsea cubeba* essential oil (LCEO) and starch with good physical properties, and investigated the effect of coating strawberries with this composite film. The best formula of the LCEO/chitosan/corn starch/glycerol (LCEO/CH/CS/gly) composite films is 0.25% LCEO, 2.75% CH, 0.40% corn starch, and 0.75% glycerol. Coating strawberries with CH/CS/gly film or LCEO/CH/CS/gly films resulted in significantly lower respiration intensity and a slower decay rate, much slower decreases in the firmness, and reductions in the sugar and ascorbic acid content of the fruit during storage (*p* < 0.05). The coatings also led to a much slower accumulation of malondialdehyde and anthocyanins (*p* < 0.05). The LCEO/CH/CS/gly film was generally more effective than the CH/CS/gly film; however, the effect was more obvious in the later stages of storage. Thus, coating strawberries with CH/CS/gly film or LCEO/CH/CS/gly film can be a viable method for extending the shelf-life of the fruit.

## 1. Introduction

Strawberry (*Fragaria × ananassa*) is a delicious and nutritious fruit, a rich source of vitamins, minerals, and natural antioxidants, and is popular with consumers [1]. Global production of strawberries was estimated at 9.2 million tons in 2016 and is forecast to reach 11.5 million tons by 2025 [2]. Strawberry is a highly perishable fruit with a short shelf-life of a few days, even when stored at refrigeration temperatures, due to the high respiration rate and the absence of peel or rind, meaning that strawberries rapidly lose water and weight [3]. In addition, the activity of enzymes such as polyphenol oxidase and peroxidase cause the degradation of anthocyanins and other polyphenols that lead to discoloration and increased darkening of the fruit’s surface; thus, strawberries are highly perishable [4]. Hence, the preservation of the postharvest quality of fresh strawberries is an urgent issue. Cold chain systems are still the best strategy for preserving strawberries, but this is quite energy-intensive and not always available or even practical in many less developed economies. Other preservation methods that have been applied include modified atmosphere packaging (MAP) and irradiation [5]. However, these methods also have drawbacks; for example, MAP can be quite expensive, and irradiation is not legally permitted in many countries. Therefore, there is still a need to develop simple, cheap, and effective methods for preserving strawberries and, indeed, other perishable fruits.

In recent years, edible coatings have gained considerable interest as a preservation method for foods, including fruits. Edible coatings can protect fruits and vegetables against mechanical, physical, chemical, and microbial damage, serving as a semipermeable barrier and thereby extending shelf life [6]. Among the many different types of edible films, chitosan (CH) film has received considerable attention. CH, a deacetylated product of chitin, is a natural polymer extracted from the shells of crustaceans and the cell walls of some algae. Due to its excellent biodegradability, biocompatibility, antimicrobial activity, and non-toxicity, CH is considered a very promising and eco-friendly material for purposes [7]. Owing to its polycationic chemical composition, CH is also a natural antimicrobial material that can inhibit the growth of many microorganisms [8]. Some studies have incorporated antimicrobial and antioxidant agents in the film to enhance the preservation effect of CH, with plant essential oil (EO) being one of the commonly used agents for this purpose [9,10]. Heras-Mozos et al. [11] reported that CH films grafted with benzaldehyde and triggered at an acidic pH controlled the in vitro growth of common fruit and vegetable spoilage and pathogenic fungi. However, the most important disadvantage of CH is its relatively high moisture permeability. Based on this concept, the combination of CH and EO represents an interesting alternative to the development of novel edible coatings.

*Litsea cubeba* (Lour.) Pers. (*Lauraceae* family), known as mountain pepper in China, is a plant that is widely distributed in Southern China, Japan, and Southeast Asia and has a long history of food use in these countries [12]. *Litsea cubeba* essential oil (LCEO) has strong antibacterial activity and has been used to improve the shelf life of foods such as vegetable juices [13].

In this work, we developed a composite CH film incorporating the LCEO and corn starch (CS) with good physical properties to explore whether they can extend the shelf-life of strawberries. Firstly, we studied the characterization and physical and mechanical properties of LCEO-CH-corn starch–glycerol (LCEO/CH/CS/gly) composite films. Secondly, we analyzed the effect of the coating on indexes related to the maturity and aging and nutritional components of strawberries.

## 2. Materials and Methods

### 2.1. Strawberry Samples and Chemicals

Strawberries were picked at market-ready maturity from the Changsha Farmers Ecological Strawberry Garden, Changsha, China. Strawberries that were regular in shape and size, weighing about 20 g each with no mechanical damage, were selected for use in the study. *Litsea cubeba* EO was extracted from mountain pepper grown in Yongshun, Hunan province, by steam distillation, and the oil contained 74.8 percent of citral, as determined by GC-MS. Chitosan, glycerol, polyvinylpyrrolidone, trichloroacetic acid, oxalic acid, and potassium ferrocyanide were purchased from Sinopharm Group Chemical Reagent Co., Ltd. (Shanghai, China) and were all analytical grade. Corn starch was purchased from Zhuzhou Xiangoose Food Company (Zhuzhou, China).

### 2.2. LCEO/CH/CS/gly Film-Forming Process

A total of 2.75% CH (*w*/*v*) was dissolved in 1.00% acetic acid (*w*/*v*) with stirring until it was completely dissolved. After that, 0.40% CS was added, followed by LCEO (1.50%, 1.75%, 2.00%, 2.25%, 2.50%, 2.75%, and 3.00%), and 1.00% gly. The mixture was manually stirred for 20 min and then stirred with a magnetic stirrer for a further 15 min. To cast the films, 40 mL of the sample solution was poured into a 150 mL culture dish, covered, and dried in a 45 °C electrothermal blast dryer for 15 h. After drying, the preservative film was carefully removed from the edge of the culture dish with tweezers and physical/mechanical property tests were performed.

### 2.3. Physical and Mechanical Properties of Films

#### 2.3.1. Thickness

The thickness of the films was measured using a micrometer caliper (Dongguan three measuring tools Co., LTD, Dongguan, China) to the nearest 0.001 mm, and the average thickness was determined at 10 random positions around the film.

#### 2.3.2. Mechanical Properties

Film samples were cut into strips of 12 cm × 6 cm [14] for tensile testing. The tensile strength (TS) and elongation at break (E) of the films were determined using the Computer Tensile Testing Machine (PNSHAR, Hangzhou, China) with an initial grip separation of 50 mm and a probe speed of 50 mm/min. The TS and E were calculated by the following equation:

$$\mathrm{TS}=\frac{F}{LW}$$

where the following hold:

F—Maximum tensile force at the film fracture, N;

L—Average thickness of the film, mm;

W—Sample width, mm.

Elongation (E%) = $\frac{L_{1}-L_{0}}{L_{0}}$where, L_1_ and L_0_ represent the length of the film after and before stretching, respectively.

#### 2.3.3. Water Vapor Permeability (WVP)

The WVP of the film was measured in accordance with Van et al. [15], with slight modifications. A small tube of 18 cm × 10 cm was used, 5 mL of distilled water was added to the test tube, and the test tube mouth was sealed with a common plastic wrap of the film, set at 25 °C and using a 90% RH chamber. The weight was accurately measured using an analytical balance and placed in a desiccator. Samples were weighed every 2 h for a total of 12 h.

$$\mathrm{WVP}=\frac{d*q}{t*S*∆P}$$

d—Sample thickness, mm;

q—Film weight gain, g;

T—Time, s;

S—Sample area, m^2^;

ΔP—Water vapor pressure difference on both sides of the film (25 °C,101 325 Pa), Pa.

#### 2.3.4. Determination of Water-Solubility (WS)

The film was cut into 40 mm × 40 mm flakes, and the weight was accurately measured using an analytical balance. It was then placed in a 50 mL conical flask filled with 30 mL of distilled water and shaken on an air shaker for 24 h. After shaking, the solution was poured it into a Petri dish, heated at 105 °C for 4 h, and accurately weighed again [16].

$$\mathrm{WS}=\frac{m_{1}-m_{2}}{m_{1}}$$

where m_1_ and m_2_ represent film weight before and after dissolution, respectively.

### 2.4. Treatment of Strawberries

**Scheme 1:**
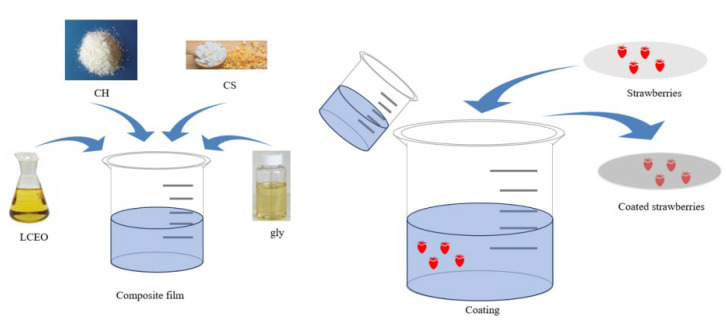
Treatment of strawberries.

Strawberries (about 6 kg) were divided into three treatment groups: the first group was immersed into the LCEO/CH/CS/gly solution for 1 min; the second and third groups were immersed into CH/CS/gly solution and sterile water (Control), respectively, for 1 min. The coated strawberries were packed in cardboard boxes and dried in an electric fan-drying oven at 25 °C for about 30 min. The strawberries treated with different coatings were further subdivided into two storage groups: (1) storage at ambient temperature (18–23 °C), RH 75%, and (2) storage at 4 °C, RH 85~90%. The physiological indices of the fruits were determined every three days. The coating process is shown in Scheme 1.

### 2.5. Measurement of Physiological Indices of Strawberries

#### 2.5.1. Decay Rate

The decay rate of the strawberries was measured using the method of Li et al. [17] in brief, the degree of decay was graded into 5 levels based on the size of the rot: Level 0—no contamination, damage or wrinkling; Level 1—decay area (infected, damaged or wrinkled) < 25% of the fruit surface; Level 2—decay area 25–50%; Level 3—decay area 50–75%; Level 4—decay area >75%. The decay rate was calculated as follows:

Decay rate (%) = ∑(decay level × number of fruits at this level)/(maximum decay level × total number of decayed fruits) × 100%.

#### 2.5.2. Firmness

The firmness of the strawberries was determined using a handheld penetrometer (GY-4, Aidebao testing instrument company, Leqing City, China). Six fruits were measured each time, and the firmness was expressed as N or kg over unit area (cm^2^).

#### 2.5.3. Weight Loss

The weight loss of strawberries was determined gravimetrically using the method of Shao et al. [18]. Since the strawberries were affected by temperature [19], weight loss was measured every 2 days for fruits stored at ambient temperature and every 3 days for those stored at 4 °C.

#### 2.5.4. Reducing Sugars

Reducing sugars were determined by the method of Lane and Eynon [20]. A brief overview s provided as follows: 

(1) Calibration of basic tartrate copper: 5 mL of basic tartrate copper solution A, 5 mL of basic tartrate copper solution B, and 10 mL of distilled water were added to a 150 mL conical flask containing two glass beads. Glucose standard solution (9 mL) was then added, and the solution was shaken well, and boiled for 2 min. Further glucose standard solution was added every 2 s until the blue color was observed to fade, and the volume consumed was recorded in triplicate.

(2) Sample treatment: A total of 5 mL of zinc acetate solution and 5 mL of potassium ferrocyanide solution were added to 2 g of strawberry puree, and the solution was diluted with water to a final volume of 250 mL. The sample was then filtered with the initial 25 mL of discarded filtrate and the remaining filtrate was retained for further testing.

(3) Filtrate pre-titration: Using the same method as Step (1), the standard solution was added quickly at the beginning, then slowly during the titration, increasing the speed when boiling until the endpoint (faded blue color). The amount of solution consumed during titration was recorded.

(4) Sample titration: The same steps as above were followed with a reduced amount of strawberry sample solution (1 mL less) compared to the pre-titration step before heating. The subsequent steps were identical, and tests were performed in triplicate. The reducing sugar content, X (g/100 g), was calculated according to the following:

X = A/(m × V/250 × 1000) × 100%

where A is the alkaline copper tartrate solution, which is equivalent to the amount of reducing sugar (mg); m is the quantity of sample (g); V is the average sample volume that was consumed (mL).

#### 2.5.5. Ascorbic Acid and Anthocyanin Content

Ascorbic acid was determined by titration with 2,6-dichlorophenol indophenol [20]. The steps are as follows: 

(1) Ascorbic acid standard solution calibration: 20 mL of ascorbic acid solution (diluted ten times with 1% oxalic acid, equivalent to 0.02 mg/mL ascorbic acid) was mixed with 0.5 mL 6% potassium iodide and 1% starch solution (3 drops). Then, the solution was titrated using 0.001 mol/L potassium iodate standard solution until the endpoint, with each standard solution tested in triplicate. The ascorbic acid (AA) concentration was calculated according to the following:

AA = V_1_ × 0.088/V_2_

where V_1_ is the volume of titrant consumed (mL); V_2_ is the amount of AA in the standard solution (mL).

(2) 2,6-Dichloroindophenol sodium calibration: 20 mL ascorbic acid solution was added to a conical flask, which was then titrated with 2,6-Dichloroindophenol sodium solution until a pink color was maintained for 15 s without fading. The volume of the titrant solution (T) was recorded, and measurements were performed in triplicate:

T = C × V_1_/V_2_

where C is the ascorbic acid concentration (mg/mL); V_1_ is the volume of 198 AA (mL); and V_2_ is the amount of 2,6-dichloroindophenol sodium that was consumed (mL).

(3) Sample solution treatment: 10 g of strawberry homogenate was weighed, 10 mL of 2% oxalic acid solution was added, and the sample solution was shaken. Then, 1% oxalic acid solution was added to a total volume of 100 mL and the solution was left to stand. White clay was added to remove the color, and the sample was filtered.

(4) Titration of sample solution: 10 mL of filtrate was collected, and the sample was titrated with a calibrated sodium 2,6-dichloroindophenol until the solution turned pink and the color was retained for 15 s. The volume of 2,6-dichloroindophenol sodium solution that was consumed was recorded and the measurements were performed in triplicate, denoting it as V_1_. In addition, 10 mL of the filtrate was promptly transferred to a 50 mL conical flask, 1 mL of 1% CuSO_4_ solution was added, and the mixture was incubated in an oven at 110 °C for 10 min. After cooling to room temperature, the sample was titrated with a standardized 2,6-dichlorophenolindophenol solution until the solution turned pink and remains unchanged for 15 s. This process was repeated three times and the average value, denoted as V_0_, was calculated. The AA content (mg/100 g) was calculated according to the following:

AA content (mg/100 g) = (V_1_ − V_0_) × T × 100/W

where V_1_ is the average amount of 2,6-dichloroindophenol that was consumed, mL; V_0_ is the average amount of 2,6-dichloroindophenol consumed by the blank space, mL; T is 1 mL of 2,6-dichloroindophenol, equivalent to the amount of AA, mg; W is the sample weight, g.

The anthocyanin content was determined using the pH differential method [21]. The steps are as follows: (1) Sample treatment: 20 mL of anthocyanin extract (acetone: formaldehyde: water: glacial acetic acid = 60 mL: 60 mL: 30 mL: 15 mL) was added to 1.5 g of strawberry homogenate and placed in a water bath at 40 °C for 4 h. The sample was then cooled to room temperature and filtered, and the filtrate was reserved for later use. (2) Determination of total anthocyanin content: 3 mL of the filtrate was mixed with 12 mL of 0.4 mol/L sodium acetate buffer (pH 4.5) in a conical flask; a further 3 mL of the filtrate was mixed with 12 mL of 0.025 mol/L potassium chloride buffer (pH 1.0) in another conical flask. After 20 min at room temperature, the absorbance of each sample was measured at 496 and 700 nm using a UV-Vis spectrophotometer.

$$\mathrm{Anthocyanin concentration}\;(mg.100^{-1}.g^{-1})=A\times MW\times DF\times 100,000\times E^{-1}$$

$$A=(A_{496},{pH}_{1.0}-A_{700},{pH}_{1.0})-(A_{496},{pH}_{4.5}-A_{700},{pH}_{4.5})$$

where MW is the molar mass (433.2 g.moL^−1^); DF is the dilution factor; and E is the absorption coefficient (15,600).

#### 2.5.6. Respiratory Intensity

Respiratory intensity was determined using the method of Tsai et al. [22]. In brief, 100 g of strawberry samples were placed in a desiccator, together with a Petri dish containing 20 mL of 0.4 M NaOH solution. After 1 h, the NaOH solution was transferred to a flask and 5 mL of saturated cesium chloride solution was added, followed by 2 drops of 1% phenolphthalein. The solution was titrated with 0.2 M oxalic acid. Respiratory intensity was calculated using the following equation:

Respiratory intensity = (V_2_ − V_1_) × M × 44 × W × H × 100%

where M is the molar concentration of the oxalic acid; V_1_ and V_2_ are the titers for the blank and sample, respectively; W is the weight of the strawberry sample; H is the time for which the respiration intensity was determined; the constant 44 represents the molecular weight of CO_2_.

#### 2.5.7. MDA

MDA content was determined according to the previous study with some modifications [23]. In brief, a sample (2 g) of strawberry was homogenized in a domestic blender and 10 mL of 7.5% trichloroacetic acid solution was added, followed by centrifugation at 10,000× *g* and 4 °C for 20 min. The supernatant was collected, 10 mL was added to 10 mL of 0.02 M thiobarbituric acid, and the mixture was heated in a boiling water bath for 20 min. The mixture was centrifuged at 10,000× *g* and 4 °C for 20 min and the absorbance of the supernatant was measured at 450, 532, and 600 nm. A blank control in which the sample was replaced by distilled water was also analyzed in the same way. The MDA concentration was calculated as follows:

MDA = [6.45 × (OD_532_–OD_600_) − 0.56 × OD_450_] × V/Vs × m × 1000 μmoL/g

where V is the volume of the extract; vs. is the volume of the sample; m is the weight of the sample; OD is the optical density at the indicated wavelength.

### 2.6. Characterization of the Coating

Scanning electronic microscopy (SEM, Jeol JSM-7610FPlus, JEOL Ltd, Tokyo, Japan) was employed to observe the surface morphology of the composite films after sputtering gold-coating; thermogravimetric analysis (TGA, Pyris-6,PerkinElmer Instruments, Shelton, CT, USA) was used to test the thermal stability of the composite films; Fourier transform infrared spectroscopy (FT-IR, Shimadzu IRTracer-100,Shimadzu Corporation, Kyoto, Japan) was used to analyze the molecular structure of the composite films.

### 2.7. Statistical Analysis

A statistical analysis of the results was performed using one-way analysis of variance and Tukey’s HSD post hoc test for means separation (significance level *p* < 0.0.5) using the statistical software IBM SPSS statistics for Windows, version 22.0. The results are reported as means with standard deviations. Unless otherwise stated, experiments were performed in triplicate.

## 3. Results and Discussion

### 3.1. Characterization of LCEO/CH/CS/gly Film

#### 3.1.1. Surface Morphology by SEM

**Figure 1:**
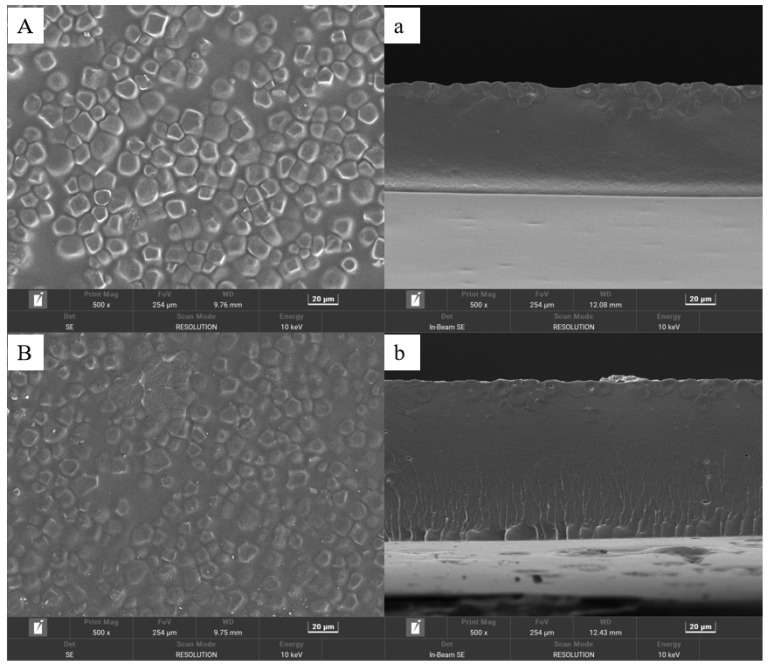
SEM of CH/CS/gly film ((**A**) film surface; (**a**) film cross-section) and LCEO/CH/CS/gly film ((**B**) film surface; (**b**) film cross-section).

In Figure 1, the SEM photographs of the surface and cross-section of the CH/CS/gly film and LCEO/CH/CS/gly film are presented. As shown in Figure 1A,B, LCEO did not change the structure of the film and is evenly distributed in the film. In Figure 1a,b, the cross-sectional structure of the film appears tight and smooth, which reflects the good film-forming performance of CH (Figure 1a). The LCEO/CH/CS/gly film is looser and pliable, which may be due to the phase separation of LCEO and film-forming base molecules, resulting in reduced compatibility (Figure 1b). This result is similar to the study by Zhao et al. [24], who reported complete and compact structures with no pores or cracks on the surface of the films. They also reported that chitosan films had a homogenous microstructure with relative roughness, whereas the cross-section and surface of the chitosan/tannic, and chitosan/tannic acid/corn starch films cross-linked by chitosan and tannic had a denser structure.

#### 3.1.2. Structural and Thermal Analysis

The infrared spectra are presented in Figure S1A. As shown in Figure S1A(c), LCEO had stretching vibration peaks at 2964 cm^−1^ and 2918 cm^−1^, which is the characteristic peak that identifies unsaturated compounds, and 1674 cm^−1^ was attributed to the stretching vibration peak of the citral carbonyl group [25]. For the CH/CS/gly film (Figure S1A(b)), the peaks at 3422 cm^−1^ and 1715 cm^−1^ correspond to OH bending vibration and CO-NH stretching vibrations [26]. In the case of the LCEO/CH/CS/gly film (Figure S1A(a)), the -CH3 and -CH2 stretching vibration peaks of citral disappear, and the peak intensity of the carbonyl group of the citral decreases significantly. This is due to the van der Waals force and hydrogen bonds between LCEO and CH/CS/gly, which suggests the successful formation of the LCEO/CH/CS/gly film. This finding is similar to the study by Go et al. [27], who reported that the addition of essential oils affects the interactions and chemical bonding between the components of chitosan composite films. Mixing the components results in an increase in the number of hydrogen bonds between the essential oils and the hydroxyl groups of chitosan, as well as interactions between the aldehyde groups in the essential oils (if present) and the amine groups of the chitosan molecules.

TGA is an effective way to study changes in the physical and chemical properties of materials [28]. Figure S1B shows the TGA thermograms of the LCEO/CH/CS/gly film (a) and CH/CS/gly film (b). In the range 30~300 °C, the mass losses of each sample show no differences. However, in the 300~600 °C range, the composite film with LCEO showed a lower mass loss rate, indicating that its thermal stability was enhanced. The results are consistent with those reported by Valencia-Sullca et al. [29], who incorporated of chitosan films with OEO oil, where the authors attributed the enhancement of the thermal stability of the films to the well-organized film matrix with a homogenous structure.

### 3.2. Physical and Mechanical Properties of Films

Table S1 shows that the addition of LCEO had little effect on the thickness of the composite films. The mechanical properties are also shown in Table S1 and, with an increase in LCEO content, the TS of composite films first increased and then decreased, with the highest TS observed at 2.25 wt%. The observed trend may be due to the enhanced intermolecular forces that occur when LCEO is below a certain level. Furthermore, the %E of the composite films showed an initial decrease followed by an increase, reaching a maximum at 3% LCEO. An increase in %E is consistent with the plasticizing effect observed with the incorporation of essential oils with many polymers. Table S1 also shows that there are no obvious trends in the WVP of the composite films with the addition of LCEO. However, at high LCEO levels, the WVP is significantly reduced due to the partitioning of LCEO on the surface of the composite films, thus reducing the WVP. The WS of composite films first increases then decreases with the addition of LCEO, with the lowest WS occurring at the lowest WVP [30]. This result is generally consistent with the study of Nguyen et al. [31], who reported that with the increase in essential oil content, the fracture elongation and water vapor transmittance of the film decreased. The main reason for this phenomenon is that the incompatibility between hydrophobic essential oil and the hydrophilic polymer (chitosan and starch) reduces the uniformity of the film structure, which is also the main reason for the decrease in TS.

### 3.3. Application of the Films in the Preservation of Strawberry

#### 3.3.1. Change in Indexes Related to Maturity and Aging

##### Change in Decay Rate

**Figure 2:**
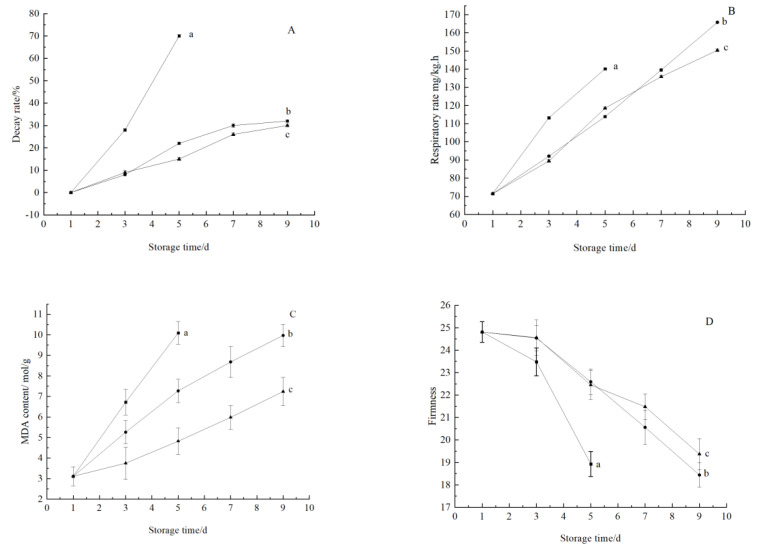
Change in indexes related to maturity and aging of strawberries with different coatings during storage at ambient temperature (25 °C). (**A**) decay rate; (**B**) respiratory intensity; (**C**) MDA concentration; (**D**) firmness. (a. control; b. CH/CS/gly film; c. LCEO/CH/CS/gly film).

**Figure 3:**
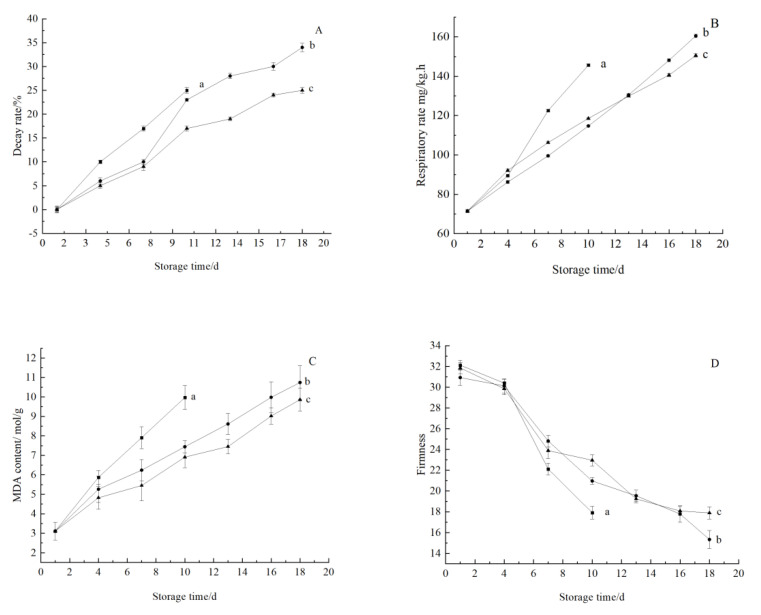
Change in indexes related to maturity and aging of strawberries with different coatings during storage at 4 °C. (**A**) decay rate; (**B**) respiratory intensity; (**C**) MDA concentration; (**D**) firmness. (a. control; b. CH/CS/gly film; c. LCEO/CH/CS/gly film).

The rate of visual decay is a simple but very useful method for evaluating the effectiveness of a preservation method for fruit, as this is the method that is most likely used by consumers to determine whether to buy or consume the fruit. As shown in Figure 2A, the decay rate of strawberries increased with the increasing duration of storage. A similar trend in the decay rate was reported by Wei et al. [32]. In Figure 2A, a significant (*p* < 0.05) variation in the decay rate can be observed compared to the control due to the application of different treatments during the observation period. The control group decayed much faster than groups coated with the CH/CS/gly film or LCEO/CH/CS/gly film, as expected, especially for the group stored at ambient temperature. After ambient storage for 3 days, about 28% of the control group decayed, and more than 70% decayed after 5 days, demonstrating that strawberry fruit is very perishable at ambient temperature without protection. In comparison, only about 8% of the CH/CS/gly film-coated strawberries or LCEO/CH/CS/gly film-coated strawberries decayed after 3 days of storage, and after 5 days, only about 22% of the treated strawberries decayed, demonstrating that the coatings were effective in protecting the fruit. There was no significant difference between the decay rate of the strawberries treated with the LCEO/CH/CS/gly film and those coated with the CH/CS/gly film (*p* < 0.05) (Figure 2A). For strawberries stored at 4 °C, the protective effect of the LCEO/CH/CS/gly film was much more significant, especially with prolonged storage. For strawberries stored at 4 °C for 7 days, the ratios of the decayed fruits were similar between those coated with the CH/CS/gly film and those with the LCEO/CH/CS/gly film, and both were much lower than the fruits treated with sterile water. After 10 days, however, about 23% of the fruit coated with the CH/CS/gly film was decayed, while for those treated with the LCEO/CH/CS/gly film, about 17% were decayed. After 18 d of storage at 4 °C, over 34% and 25% of the strawberries were decayed when coated with the CH/CS/gly film and the LCEO/CH/CS/gly film, respectively (Figure 3A).

These results were not surprising, as mold contamination was the main cause of strawberry decay during storage, and it is well documented that CH has an inhibitory effect on microorganisms, including molds. Furthermore, LCEO has been shown to have significant antimicrobial effects [33]. Its inclusion in the composite film would enhance the protection of the strawberries against mold contamination and retard the decay of the fruit. Similar effects have been reported by Zheng et al. [34].

##### Changes in Respiratory Intensity

Respiration is one of the most important physiological activities occurring in fruits and vegetables and is perhaps the most crucial factor in determining their postharvest shelf-life [35]. Moreover, respiration intensity is reported to continue to increase during storage [36]. Figure 2B shows the changes in the respiratory rate of strawberries treated with different coatings during storage, with a significant variation (*p* < 0.05) found in relation to the respiratory intensity that occurs at different days of storage due to the different treatments. Among the different treatments, the respiratory intensity of the control group was significantly higher (*p* < 0.05) as compared to fruits in other treatments. Irrespective of the treatments, the respiration rate of the strawberries continuously increased during storage, a phenomenon that has been well-documented in the literature [37]. However, the increase was much faster in the control than in those coated with the CH/CS/gly film and the LCEO/CH/CS/gly film. For example, for strawberries stored at ambient temperature, the respiration rate was about 113.28 mg/kg·h after 3 d for the control group, compared with about 90.00 mg/kg·h for those coated with the CH/CS/gly film and LCEO/CH/CS/gly film. The respiration rate between strawberries coated with the CH/CS/gly film and LCEO/CH/CS/gly film was not significantly different in the first 5 days of storage. With further storage, however, the respiration rate increased faster in the former than the latter, and by 9 d, it was 165.78 mg/kg·h for the former and 150.42 mg/kg·h for the latter (Figure 2B). For storage at 4 °C, the differences between the respiration rate of the fruits with different treatments were not significant during the first 4 d of storage. However, thereafter, the respiration rate increased much faster in the control than in the samples coated with the CH/CS/gly film and LCEO/CH/CS/gly film. By 10 d, the respiration rate of the control group was 145.64 mg/kg·h, compared to about 114.72 mg/kg·h and 118.54 mg/kg·h for the samples coated with the CH/CS/gly film and LCEO/CH/CS/gly film, respectively. Similar to storage at ambient temperature, the respiration rates of the CH/CS/gly film-coated fruits and LCEO/CH/CS/gly film-coated fruits were not substantially different in the early to mid-stages of storage at 4 °C. After 13 d, the respiration rate of CH/CS/gly film-coated fruits increased faster than that of LCEO/CH/CS/gly film-coated fruits. By 18 d, the respiration rate in the former was 160.52 mg/kg·h, compared with 150.54 mg/kg·h for the latter (Figure 3B).

The lower respiration rates in chitosan-coated strawberries were likely due to the low permeability of the film to O_2_ and CO_2_ [38], which could induce a low O_2_ and high CO_2_ environment immediately surrounding the fruit, thus retarding its physiological activities including respiration. It is not clear why the LCEO/CH/CS/gly film retarded the respiration rate more than the CH/CS/gly film. Possible reasons for this include interactions of the EO components with O_2_ and ethylene, and the antimicrobial activities of the EO, which suppressed mold growth, which is known to accelerate respiration in infected fruits [33].

##### Changes in MDA Concentration

MDA is a natural product of membrane lipid peroxidation, which can aggravate the damage to cell membranes [39]. It is a common indicator used in the study of the postharvest physiology of fruits and vegetables. Increases in MDA concentration indicate membrane oxidation and aging [13]. Figure 2C and Figure 3C show the effect of the three different treatments on the MDA concentration of strawberries during storage at ambient temperature and 4 °C. MDA concentration continuously increased during storage under both storage conditions. There were significant differences among the different treatments regarding the MDA concentration when compared with the control (*p* < 0.05). The increase in MDA concentration was fastest in the control, followed by fruits coated with the CH/CS/gly film while the increase was the slowest in samples coated with the LCEO/CH/CS/gly film. This trend was consistent for storage at both ambient temperature and 4 °C, although the increases were faster at the higher temperature, as expected. For storage at ambient temperature, the MDA concentration at day 5 was 10.09 mol/g, 7.27 mol/g, 4.82 mol/g for the control group, CH/CS/gly film-coated fruits, and LCEO/CH/CS/gly film-coated fruits, respectively. For fruits stored at 4 °C (Figure 3C), the accumulation of MDA was much slower, and after only 18 d of storage, its concentration reached 10.74 and 9.86 μmoL/g for CH/CS/gly film-coated fruits and LCEO/CH/CS/gly film-coated fruits, respectively. The much slower accumulation of MDA was most likely due to the incorporation of LCEO in the coating mixture, as it is well documented that LCEO, like most EOs, has strong antioxidant activities [40], which would retard the oxidation of the lipids in the cell membrane.

The slower accumulation of MDA in fruits stored at 4 °C is expected, as the oxidation of lipids occurs at a slower rate when the temperature is decreased [41]. Moreover, Xiao et al. [42] reported that the chitosan/zein–cinnamaldehyde coating treatment significantly slowed down the accumulation rate of MDA, which could be attributed to the free radicals’ scavenging potential and protective effects on the cell membrane of CH. Obviously, uncoated strawberries had a significantly higher (*p* < 0.05) MDA content than coated strawberries. In addition, the data for MDA in coated strawberries fluctuated at the early stage of storage in the present study, which may be due to the CH coating, which has a certain ability to repair the membrane peroxidation of strawberries.

##### Changes in Firmness

Firmness is one of the major quality indicators for strawberries, as it affects the appearance and texture of the fruit. Firmness declines during storage as endogenous pectinases break down pectin, which is the main component responsible for the structural integrity of fruits [43]. Figure 2D and Figure 3D show changes in the firmness of strawberries treated with different coatings. Significant variations in firmness were observed compared to the control due to the application of different treatments during the observation period (*p* < 0.05). The control showed the fastest decreases in firmness at both storage temperatures, while decreases in firmness were much slower for the fruits treated with either chitosan or the chitosan–EO composite film. For example, at ambient temperature, the firmness of strawberries treated with sterile water decreased from 24.81 kg/m^2^ to 18.93 kg/m^2^ after 5 d, whereas the firmness decreased to about 22.00 kg/m^2^ for the fruits coated with the CH/CS/gly film and LCEO/CH/CS/gly film. The firmness of the fruits treated with the LCEO/CH/CS/gly film was slightly higher than those coated with the CH/CS/gly film throughout the storage period, but the differences were not substantial and probably not commercially significant (Figure 2D). Similar trends were also observed for strawberries stored at 4 °C, although it took about twice as much time for the firmness to reach the levels of fruits stored at ambient temperature (Figure 3D).

The slower decreases in CH/CS/gly film-coated strawberries are most likely due to the modified atmosphere, where a low O_2_ and high CO_2_ environment was created due to the low gas permeability of the CH/CS/gly film. As discussed above, such an environment led to a decrease in respiration rate, and possibly the overall physiological activities of the fruit, with a consequently slower softening of the fruit. The ability of MAP to retard the softening of strawberries has been reported in the literature [44]. Fresh strawberries, a perishable fruit, have a very high respiration rate at 20 °C and are also very sensitive to the development of molds. The small further improvement in slowing down the softening of strawberries obtained using the composite film could be due to its effects in lowering the respiration rate, as discussed above.

#### 3.3.2. Change in Nutritional Components

**Figure 4:**
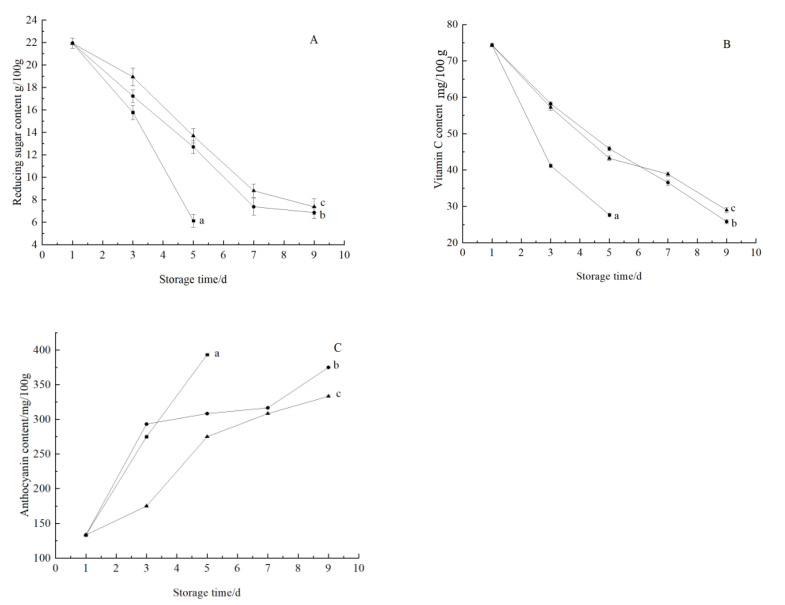
Change in the nutritional components of strawberries with different coatings during storage at ambient temperature (25 °C). (**A**) reducing sugars; (**B**) ascorbic acid; (**C**) anthocyanin. (a. control; b. CH/CS/gly film; c. LCEO/CH/CS/gly film).

**Figure 5:**
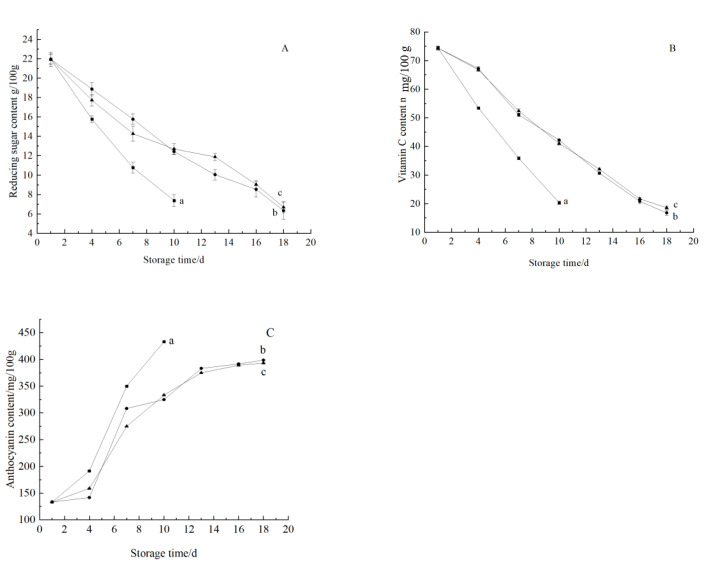
Change in nutritional components of strawberries with different coatings during storage at 4 °C. (**A**) reducing sugars; (**B**) ascorbic acid; (**C**) anthocyanin. (a. control; b. CH/CS/gly film; c. LCEO/CH/CS/gly film).

Reducing sugars, ascorbic acid, and anthocyanin levels are important components of strawberries due to their taste and nutritional quality. Figure 4 and Figure 5 show changes in the concentration of reducing sugars, ascorbic acid, and anthocyanin, respectively, during the storage of strawberries treated with different coatings. The concentration of reducing sugars decreased during storage for all strawberry samples; however, the rate of the decrease was significantly faster in the control group than those coated with the CH/CS/gly film and LCEO/CH/CS/gly film (*p* < 0.05). At an ambient temperature, the reducing sugar concentration in the control decreased from 21.94 to 6.14 g/100 g in 5 d, while during the same period, it only decreased to 12.71 g/100 g and 13.69 g/100 g for the CH/CS/gly film-coated fruits and LCEO/CH/CS/gly film-coated fruits, respectively. The concentrations of reducing sugars in CH/CS/gly film-coated samples and LCEO/CH/CS/gly film-coated samples were not significantly different in the initial 5 d; after that, however, the decrease in the former was significantly faster than that in the latter (Figure 4A). The same trend was observed for storage at 4 °C, although the decrease in reducing sugars was much slower at this temperature and the concentration of reducing sugars in CH/CS/gly film-coated fruits was not significantly different from that of the LCEO/CH/CS/gly film-coated samples until the last days of storage (Figure 5A). The slower decreases in reducing sugars in the CH/CS/gly film-coated strawberries and LCEO/CH/CS/gly film-coated strawberries were likely due to the lower respiration rates of the fruits, as discussed above. A similar trend in the decay rate has been reported in the literature [45,46], where the rates of decay of anthocyanins, reducing sugars, and polyphenols in freshly cut aubergines wrapped in chitosan film was lower than that of the control group [46]. Moreover, the use of a chitosan composite film can effectively inhibit the decline in strawberry anthocyanin and other contents [45]. This may be due to the fact that a chitosan composite film can increase the gas concentration and humidity inside the package, reduce the condensation of water vapor on the surface, and inhibit the activity of related enzymes.

Strawberry fruit is rich in ascorbic acid, but the level of this acid decreases during postharvest storage due to oxidation [47]. The ascorbic acid content in the strawberries declined rather rapidly during storage, especially when stored at ambient temperature, but the decline was significantly slower in chitosan- and composite-coated samples than in the control (*p* < 0.05). For example, at ambient temperature, the ascorbic acid content in the control declined from 74.35 to 27.65 mg/g in 5 d, while it declined to 45.87 and 43.22 mg/g for the CH/CS/gly film-coated samples and LCEO/CH/CS/gly film-coated samples, respectively, for the same period. The differences between the ascorbic acid content of CH/CS/gly film-coated fruits and LCEO/CH/CS/gly film-coated fruits were relatively small, especially for samples stored at 4 °C. The protective effects of the CH/CS/gly film coating and LCEO/CH/CS/gly film coating against ascorbic acid loss were probably attributable to their low gas permeability, which provides a low-O_2_ environment that retards the oxidation of ascorbic acid [16].

Anthocyanins are flavonoids with a strong antioxidant capacity, and the consumption of fruits and vegetables rich in anthocyanins has been reported to confer a number of health benefits [48]. Berry fruits such as strawberries are a rich source of anthocyanins in the diet. Figure 4C shows the changes in anthocyanin content during the storage of strawberries treated with different coatings. The concentration of anthocyanins increased continuously during storage in all strawberry samples, and this increase was fastest in the control group, especially during the later stages of storage, followed by the increase in the CH/CS/gly film-coated fruits, while the increase was slowest in the LCEO/CH/CS/gly film-coated samples (*p* < 0.05). For samples stored at ambient temperature, the concentration of anthocyanins in the control group increased almost linearly and reached 393.21 mg/100 g at 5 d. During the same period, its concentration in chitosan- and composite-coated fruits increased to 308.24 and 274.92 mg/100 g, respectively (Figure 4C). For strawberries stored at 4 °C, the concentration of anthocyanins in the control group increased very fast throughout the 9 days of storage, while the increase was also fast (although slower than the control) in CH/CS/gly film-coated fruits and LCEO/CH/CS/gly film-coated fruits during the initial 7 days, but equilibrated to some extent after that. There were no significant differences between the concentrations of anthocyanins in CH/CS/gly film-coated fruits and LCEO/CH/CS/gly film-coated fruits (Figure 5C). It is generally known that the concentration of anthocyanins increases during the maturation of fruits, including strawberries, which can continue after storage [49]. Higher concentrations of O_2_ are generally favorable to the accumulation of anthocyanins during the early stages of storage [50]. Thus, the slower increases in the concentration of anthocyanins in CH/CS/gly film-coated strawberries and LCEO/CH/CS/gly film-coated strawberries compared to the control group were likely a result of the restricted access to O_2_ due to the low gas permeability of the films.

## 4. Conclusions

In this study, we demonstrated that coating strawberries with CH/CS/gly and LCEO/CH/CS/gly films was effective in slowing down their postharvest physiological activities and extending their shelf-life during storage at ambient temperature and 4 °C. Compared to the control group (coated with water), CH/CS/gly film-coated strawberries and LCEO/CH/CS/gly film-coated strawberries had significantly lower respiration intensities and decay rates, and their firmness was much greater. The coated fruits also had a much slower accumulation of MDA during storage, indicating that they suffered less lipid peroxidation in the cell membrane. Furthermore, the coated strawberries also retained more reducing sugars and ascorbic acid during storage. The LCEO/CH/CS/gly film was generally more effective than the CH/CS/gly film; however, the effects were more obvious in the later stages of storage.

## Data Availability

The original contributions presented in the study are included in the article/Supplementary Material, further inquiries can be directed to the corresponding author/s.

## Supplementary Materials

The following are available online at https://www.mdpi.com/article/10.3390/foods13040599/s1, Figure S1: FTIR spectra (A) and TGA analysis (B) of the LCEO/CH/CS/gly film (a), CH/CS/gly film (b) and LCEO (c), Table S1: Effect of LCEO on mechanical properties of composite films.
