# Supplementary material for: Effects of Litsea cubeba Essential Oil–Chitosan/Corn Starch Composite Films on the Quality and Shelf-Life of Strawberry (Fragaria × ananassa)
Source: Foods. 2024 Feb 16;13(4):599. doi: 10.3390/foods13040599 (PMC10888304; doi:10.3390/foods13040599)
Supplement: Supplementary file 1 [file foods-13-00599-s001.zip › foods-2833630-supplementary.pdf]

Effects of *Litsea cubeba* Essential Oil–Chitosan/Corn Starch Composite Films on the Quality and Shelf-Life of Strawberry (*Fragaria* × *ananassa*)

Hongjun Fu [1,†], Liyuan Wang [1,†], Jiahui Gu [1], Xianglian Peng [1,*] and Jian Zhao [2,*]

[1] College of Food Science and Engineering, National Engineering Laboratory for Deep Process of Rice and Byproducts, Central South University of Forestry and Technology, Changsha 41004, China.

[2] Food Science and Technology, School of Chemical Engineering, UNSW Australia, Sydney 2052, Australia

[†] These authors contributed equally to this work

* Correspondence: xianglianpeng@csuft.edu.cn (X.P.); jian.zhao@unsw.edu.au (J.Z.); Tel.: +86-731-85623240 (X.P.); +61-2-9385-4304 (J.Z.)

Table S1. Effect of LCEO on mechanical properties of composite films

LCEO（%）	Thickness (mm)	TS (MPa)	E (%)	WVP (mg $H_2O\cdot m/h\cdot m^2$)	WS (%)
1.50	0.072±0.003	4.444±1.840^{d}	51.00±5.840^{b}	0.723±0.023^{d}	0.1288±0.002^{e}
1.75	0.059±0.002	10.200±0.603^{b}	44.44±2.210^{b}	0.901±0.023^{a}	0.1432±0.003^{d}
2.00	0.062±0.010	10.645±2.138^{b}	42.44±1.002^{b}	0.523±0.008^{b}	0.1585±0.002^{d}
2.25	0.065±0.003	24.877±1.212^{a}	67.88±1.692^{a}	0.601±0.005^{c}	0.2224±0.001^{b}
2.50	0.063±0.007	13.762±0.611^{b}	70.08±1.422^{a}	0.723±0.019^{d}	0.2521±0.001^{a}
2.75	0.068±0.017	5.059±0.493^{d}	77.23±1.191^{d}	0.401±0.027^{a}	0.1820±0.007^{c}
3.00	0.068±0.007	7.265±0.529^{c}	79.01±1.442^{d}	0.423±0.018^{a}	0.0553±0.004^{f}

Notes: Values are means ± standard deviations. a is the maximum or minimum average; b, c, d, e and f indicate significant difference ($P<0.05$).

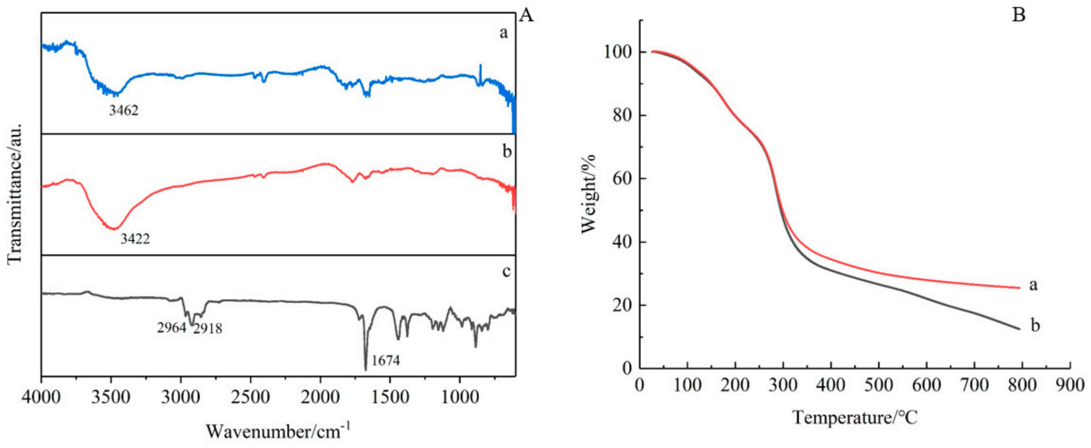

Figure S1. FTIR spectra (A) and TGA analysis (B) of the LCEO/CH/CS/gly film (a), CH/CS/gly film (b) and LCEO (c)
